# The Expression Profile, Clinical Application and Potential Tumor Suppressing Mechanism of hsa_circ_0001675 in Head and Neck Carcinoma

**DOI:** 10.3389/fonc.2022.769666

**Published:** 2022-05-06

**Authors:** Yujie Cao, Dong Ye, Zhisen Shen, Zan Li, Qun Li, Hao Rong

**Affiliations:** ^1^ Department of Otorhinolaryngology Head and Neck Surgery, Lihuili Hospital Affiliated to Ningbo University, Ningbo, China; ^2^ Department of Otorhinolaryngology Head and Neck Surgery, Ningbo Medical Center Lihuili Hospital , Ningbo, China; ^3^ Medical School of Ningbo University, Ningbo, China; ^4^ The Affiliated Cancer Hospital of Xiangya School of Medical, Central South University, Changsha, China

**Keywords:** HNC, circRNA, microRNA, hsa_circ_0001675, hsa-miR-577, TESC, bioinformatics

## Abstract

**Purpose:**

This study sought to identify circular RNAs (circRNA) that participate in the regulation of head and neck cancer (HNC), analyze their clinical application, and predict their molecular mechanism during HNC.

**Materials and Methods:**

High-throughput sequencing was used to analyze circRNA expression in 18 matched HNC and adjacent normal tissues. Target circRNAs with significantly differential expression were obtained. In 103 HNC and adjacent normal tissues, real-time fluorescent quantitative PCR (qRT-PCR) was used to verify the differential expression of target circRNAs. This data was combined with clinicopathological information to analyze the diagnostic value of target circRNA. Bioinformatics was used to find target circRNAs that acted as competitive endogenous RNA (ceRNA) and construct a circRNA-miRNA-mRNA regulatory network. mRNA expression was verified by immunohistochemistry (IHC).

**Results:**

A total of 714 differentially expressed circRNAs were detected in HNC, and the low expression of hsa_circ_0001675 was particularly significant (fold change [FC] = -4.85, *P* = 6.305E-05). hsa_circ_0001675 had significantly lower expression in HNC than in normal tissue (*P* < 0.01). Low hsa_circ_0001675 expression was positively associated with tumor invasion and clinical staging (*P* < 0.05), and its area under the ROC curve (AUC) was 0.7776. Low hsa_circ_0001675 expression also correlated with the overall survival (OS) rate and the progression-free survival (PFS) rate of HNC patients (*P* < 0.001). Bioinformatics was used to construct a ceRNA network of hsa_circ_0001675 with six differentially expressed miRNAs (hsa-miR-330-5p, hsa-miR-498, hsa-miR-532-3p, hsa-miR-577, hsa-miR-1248, and hsa-miR-1305) and 411 differentially expressed mRNAs and found that the neuroactive ligand-receptor interaction, and the cAMP and calcium signaling pathways were particularly enriched. Further bioinformatics and IHC analysis showed that miR577/TESC is the likely downstream signaling pathway for hsa_circ_0001675.

**Conclusion:**

This study showed that hsa_circ_0001675 is downregulated in HNC and could be an effective biomarker for HNC diagnosis. In addition, hsa_circ_0001675 may have a potential ceRNA mechanism and suppress HNC disease progression through the hsa_circ_0001675-miRNA-mRNA axis.

## Introduction

Head and neck cancer (HNC) includes tumors in the nasal cavity, oral cavity, pharynx, sinuses, and other head and neck tissues. It is the world’s sixth most common cancer, with more than 600,000 new cases diagnosed each year. Head and neck squamous cell carcinoma (HNSC) accounts for 95% of all HNCs. Primary factors for HNC include smoking, excessive alcohol consumption, and infection with human papillomavirus (HPV) (1). Most patients present locoregionally advanced disease, which is characterized by tumor heterogeneity, local regional metastasis, and resistance to existing treatments (2). Occult cervical lymph node metastases can occur even in early-stage head and neck cancer (3). The overall survival (OS) of patients with recurrence and metastasis is about six months, and the prognosis is not ideal (4). Therefore, there is an urgent need for accurate biomarkers for the diagnosis and prognosis prediction of HNC patients, and to improve the survival rate (5). HNC treatment is complex but new approaches are emerging to supplement traditional surgical treatment, including targeted therapy using small molecule inhibitors or antibodies, immunotherapy, and combination therapy (6). However, the five-year OS rate of HNSC is currently about 50%, indicating that treatments remain unsatisfactory (7). Poor outcomes are usually the result of late-stage diagnosis and ineffective treatment (8). Therefore, it is also necessary to develop safe and effective methods of treatment for HNC patients.

Circular RNA (circRNA) is a covalently closed, single-stranded RNA molecule, produced by reverse splicing of pre-mRNAs and is a member of the non-coding RNA (ncRNA) class. CircRNA expression plays a significant role in the occurrence and development of HNC, but the mechanisms by which it impacts disease are not fully understood. Since HNC lacks effective early diagnostic markers and new clinical treatments, this study used high-throughput sequencing and real-time fluorescent quantitative PCR (qRT-PCR), combined with HNC-specific clinical data and information from The Cancer Genome Atlas (TCGA) database, to identify new circRNAs with high diagnostic value. Bioinformatics was used to analyze related signaling pathways and explore potential circRNAs-miRNA-mRNA regulatory pathways to better understand the pathogenesis of HNC and provide a scientific basis for early clinical diagnosis and targeted treatment.

## Materials and Methods

### Specimen Collection

All paired HNC and adjacent normal non-tumor tissue samples (dissected at >0.5 cm from the edge of the tumor lesion) were collected from 121 patients at the Department of Otolaryngology Head and Neck Surgery of Ningbo Medical Center in Li Huili Hospital and Hunan Cancer Hospital from 2012 to 2019. All tissue samples were blindly examined by two or more pathologists and confirmed to be tumor or adjacent normal non-tumor tissue. All tissue samples were transferred to an RNA protection solution immediately after surgery and stored at –80°C until use. Tumor staging was conducted according to the American Joint Committee on Cancer (AJCC) tumor-node-metastasis (TNM) staging system (7th Edition). No patients had received any chemotherapy or radiotherapy prior to surgery. Preoperative ultrasound, computed tomography (CT), and other tests confirmed that all cases were primary HNC. Finally, the study obtained informed consent from all patients and was approved by the Research Ethics Committee of Ningbo University. The number of the ethics review approval document is KY2020PJ100. A total of 18 paired HNC samples were used for high-throughput sequencing analysis, and 103 paired HNC samples were used to verify differences in circRNA expression.

### RNA Extraction and Reverse Transcription

Total RNA was extracted from the HNC samples and adjacent non-tumor normal tissues using Trizol reagent (Invitrogen Life Technologies, CA, USA). RNA purity and concentration were measured using a NanoPhotometer spectrophotometer (IMPLEN, CA, USA), and Qubit^®^3.0 Fluorometer (Life Technologies, CA, USA). The RNA Nano 6000 Assay Kit from the Bioanalyzer 2100 system (Agilent Technologies, Santa Clara, CA, USA) was used to evaluate RNA integrity. The optical density (OD) at 260 nm (A260 = 1 for 40 ug/mL RNA) was used to determine the quantity of RNA, while the ratio of the ODs obtained at 260 and 280 nm (A260/280) was used to assess RNA purity, which was controlled at a range of 1.8–2.1. The transcription kit (Promega, Madison, WI, USA) was used for reverse transcription.

### Quantitative Real-Time Polymerase Chain Reaction

Sequences of the circRNA and human GAPDH (housekeeping gene) were obtained from the NCBI GenBank database (NCBI, http://www.ncbi.nlm.nih.gov/) and the PCR primers were designed by Primer5.0 and Primer3web (https://primer3.ut.ee/). Primer sequences used for quantitative real-time polymerase chain reaction (qRT-PCR) included: hsa_circ_0001675: sense, TCAACCTACCTTACCTGAACGT, and antisense, GGATTTAGAGGCCATTTCCCG; and GAPDH sense, CATGAGAAGTATGACAACAGCCT, and antisense, AGTCCTTCCACGATACCAAAGT. The cDNA obtained by reverse transcription were used as templates, and the thermocycling parameters included: 95°C for 10 minutes followed by 40 cycles of 95°C for 10 minutes, 60°C for 30 seconds, and 72°C for 1 second. The amplification reaction was carried out on the Stratagene MX3005P Real-time PCR machine according to the standard protocol using Go Taq^®^ qPCR Master Mix kit (Promega, Madison, WI, USA). The Ct value represented the number of reaction cycles achieved when the intensity of the PCR fluorescence signal reached the fluorescence threshold set by the machine and was used to indicate the template quantity. The housekeeping gene, GAPDH, was used to obtain a quantitative calculation of the target circRNA using standard reference material. RT-PCR results are expressed as ΔCt to quantitatively analyze circRNA levels. The larger the ΔCt value, the lower the expression of circRNA. Biological analysis of each sample was performed in triplicate.

### Target mRNA Screening Process of hsa_circ_0001675

By using the R “limma” package, we obtained miRNAs expression data of head and neck squamous cell carcinoma (HNSC) from the Cancer Genome Atlas (TCGA) database and screened the differentially expressed miRNAs. The circular RNA Interactome database (https://circinteractome.irp.nia.nih.gov/) was used to predict the target miRNAs for hsa_circ_0001675. By taking the intersection of differentially expressed miRNAs in the TCGA database and predicted miRNAs in the circular RNA Interactome database, we obtained the target miRNAs. The TargetScan database (https://www.targetscan.org/) and miRDB database (https://www.mirdb.org/) was used to predict the target mRNAs, and R “limma” package was used to screen mRNAs that was differentially expressed in HNSC in the TCGA database. The intersection of the three prediction results was used to define the target mRNAs. Clinical data of HNSC (n=519) were also downloaded through TCGA and used by the R “survival” package to analyze the correlation between the obtained target mRNA and overall survival time in HNSC.

### Immunohistochemistry Staining and Analysis

The surgically resected tumor and adjacent tissues were fixed with formalin, embedded in paraffin, cut into 4 μm-thick sections, and deparaffinized with xylene and ethanol. The slices were immersed in sodium citrate buffer (pH 6.0), boiled at 120°C for 20 minutes for antigen retrieval, and incubated in 3% H_2_O_2_ at room temperature for 10 minutes to block endogenous peroxidase. For immunohistochemistry (IHC), the sections were incubated with TESC rabbit monoclonal antibody (ProteinTech, Cat No.11125-1-AP) followed by incubation with the secondary antibody. The sections were developed with diaminobenzidine (Sigma, Cat No. ZLI-9018) solution, counterstained with hematoxylin, and examined under a microscope after dehydration with various concentrations of alcohol. TESC immune response sections were observed under a microscope and interpreted by qualified pathologists.

### Data Processing Analysis

Statistical analysis and image processing of all data was performed using the IBM SPSS software version 22.0 (IBM Corp, Armonk, NY, USA) and GraphPad Prism version 9.0 (GraphPad Software, San Diego, CA, USA). The normality of data was assessed by the Shapiro–Wilk test. A paired-sample T test was used to determine differences in hsa_circ_0001675 expression in tumor tissues and paired normal tissues. The Chi-square test and Fisher exact test were used to identify the differences between HNC clinical features, including gender, age, primary site, differentiation type, clinical stage, lymph node metastasis, smoking history, and drinking history, and hsa_circ_0001675 expression. Receiver operating characteristic curves (ROC) were constructed to analyze the value of hsa_circ_0001675 in the diagnosis of HNC. The mean of ΔCt values of qrt-PCR was used to distinguish high and low expression of hsa_circ_0001675. The Kaplan-Meier method was used to estimate OS and PFS of patients with high or low expression of hsa_circ_0001675, and the cox proportional hazards model was used for multivariate analyses of prognostic factors. The hazard ratio (HR) of each variable was determined by its 95% confidence interval (CI). A P-value <0.05 was considered statistically significant.

## Results

### High-Throughput Sequencing

#### Analysis of Differential circRNA Expression

**Figure 1:**
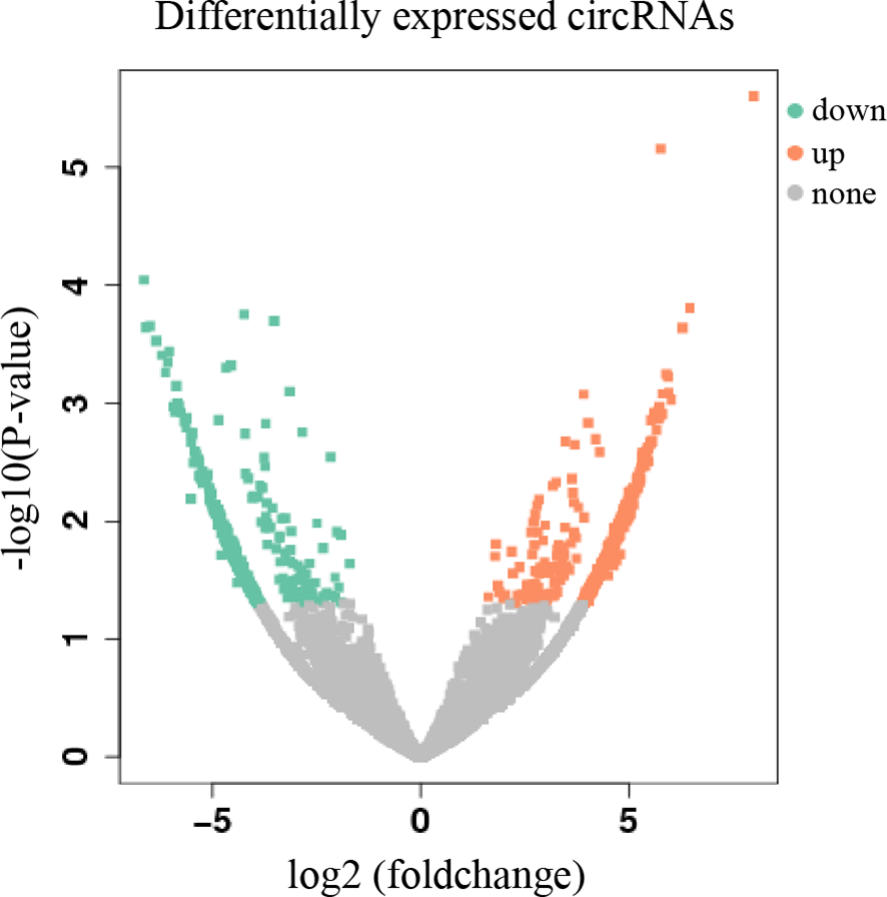
Volcano plot of differentially expressed circRNAs. The abscissa is log2 (foldchange), and the ordinate is -log10 (p-value). The orange dots in the upper right corner indicate up-regulated genes, and the green dots in the upper left corner indicate down-regulated genes. The grey dots at the bottom represent genes that were not significantly differentially expressed.

High-throughput sequencing was used to construct the circRNA expression profile from tumor and adjacent tissues from 18 HNC patients. A total of 40,577 circRNAs were detected. Sequencing results indicated that 714 circRNAs were differentially expressed in tumor and adjacent normal tissues (log2 (foldchange) >1.5, *P* < 0.05), of which 382 were upregulated and 332 were downregulated (**Figure 1**).

### Differentially Expressed circRNA and qRT-PCR Verification

**Figure 2:**
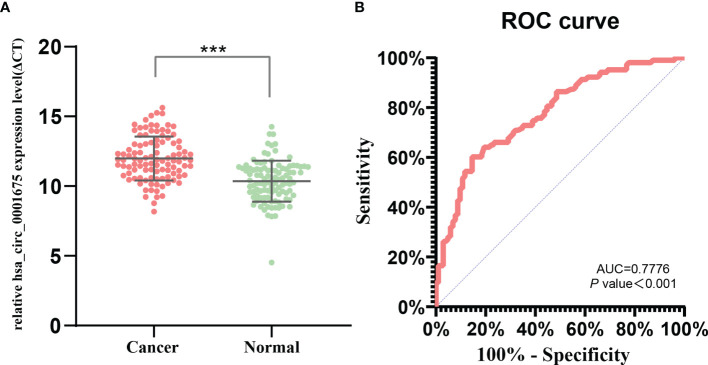
hsa_circ_0001675 expression and diagnostic value. **(A)** hsa_circ_0001675 was significantly downregulated in 103 paired HNC samples. **(B)** Receiver operating characteristic (ROC) curve of hsa_circ_0001675. ***P < 0.001.

Expression of hsa_circ_0001675 was significantly downregulated in tumor tissues (foldchange = -4.85, *P* = 6.305E-05) and was chosen for subsequent qRT-PCR verification. QRT-PCR of 103 matched HNC samples confirmed that hsa_circ_0001675 had a higher ΔCt value in tumor tissues, indicating that it had lower expression in tumor than in normal adjacent tissues (*P* = 1.6152E-15) (**Figure 2A**).

### Correlation Between hsa_circ_0001675 Expression and the Clinicopathological Characteristics of HNC Patients

Expression of hsa_circ_0001675 is related to the degree of tumor invasion. hsa_circ_0001675 expression was lower in patients with advanced T-stage tumors (Tis/T1/T2 *vs* T3/T4, *P*=0.001) and in advanced clinical patients (I/II *vs* III/IV, *P*<0.001). However, differences in expression of hsa_circ_0001675 were not significantly associated with gender, age, smoking behavior, alcohol behavior, tumor site, histologic grade, tumor invasion, lymphatic metastasis, or clinical stage (**Table 1**).

**Table 1:** The relationship between the expression level of hsa_circ_0001675 and clinicopathological characteristics of HNC patients.

Characteristics		hsa_circ_0001675 expression		*P* value
		high	low	
**Gender**				
male	102	51	51	1.000
female	1	1	0	
**Age**				
<60y	37	23	14	0.076
≥60y	66	29	37	
**Smoking behavior**				
No	42	23	19	0.471
Yes	61	29	32	
**Alcohol behavior**				
No	46	22	24	0.628
Yes	57	30	27	
**Tumor site**				
Oral cavity/Pharynx	23	11	10	0.846
Larynx	80	41	41	
**Histologic grade**				
Well/Moderately	67	35	31	0.407
Poorly	36	16	20	
**Tumor Invasion**				
Tis/T1/T2	47	32	15	0.001
T3/T4	56	19	37	
**Lymphatic metastasis**				
No	67	38	29	0.084
Yes	36	14	22	
**Clinical stage**				
I/II	42	30	12	<0.001
III/IV	61	22	39	

P value indicates statistical significance.

### The Diagnostic Value of hsa_circ_0001675 for HNC

Using normal adjacent tissue as a control, a ROC was constructed to analyze and evaluate the diagnostic value of hsa_circ_0001675 for HNC (**Figure 2B**). The area under the ROC curve (AUC) of hsa_circ_0001675 was 0.7776 (95% CI = 0.7151–0.8402, *P* < 0.001), indicating that this circRNA had a high diagnostic value. The cut-off point was ΔCt = 11.51 and the sensitivity and specificity were 60.19% and 85.44%, respectively.

### Correlation Between hsa_circ_0001675 Expression and HNC Patient Survival

**Figure 3:**
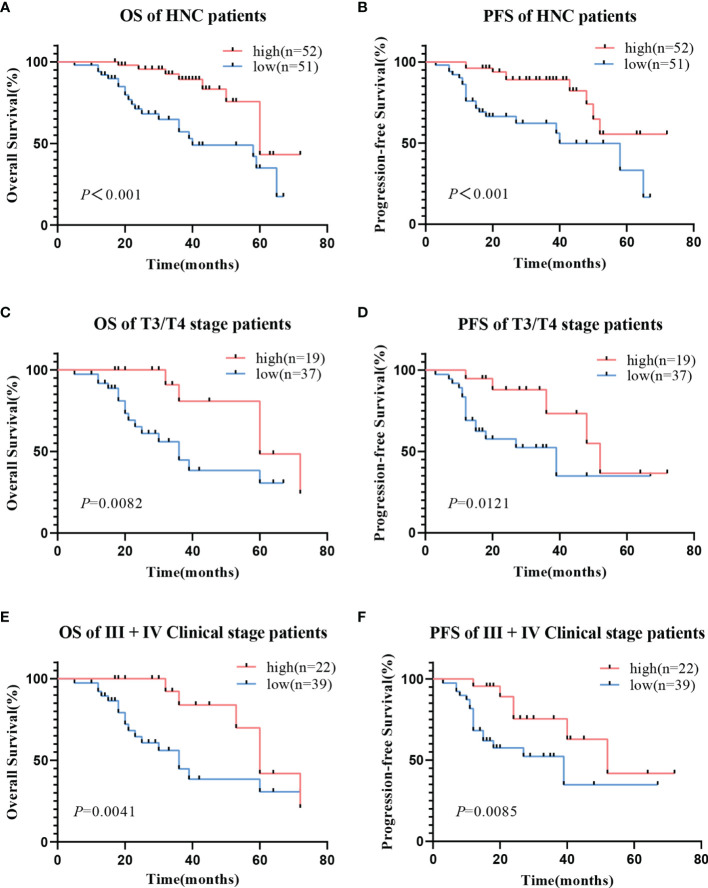
Relationship between hsa_circ_0001675 expression and OS rate and PFS rate of HNC patients. **(A)** Low hsa_circ_0001675 expression was related to poor OS. **(B)** Low hsa_circ_0001675 expression was related to poor PFS. **(C, D)** Low hsa_circ_0001675 expression was significantly correlated with poor OS and poor PFS in advanced T stage (T3/T4) patients. **(E, F)** Low hsa_circ_0001675 expression was significantly correlated with poor OS and PFS in advanced clinic stage patients (III, IV).

Kaplan-Meier analysis showed that low hsa_circ_0001675 expression was negatively correlated with the OS and PFS of HNC patients (log-rank *P*<0.001) (**Figures 3A, B**). Based on clinical factors like tumor invasion and clinical stage that were significantly associated with hsa_circ_0001675 expression, a subgroup analysis was conducted for OS and PFS. Low hsa_circ_0001675 expression did not correlate significantly with OS in early T stage (Tis/T1/T2) patients but was associated with lower OS in advanced T stage (T3/T4) patients (log-rank *P* = 0.0082) (**Figure 3C**). Results also showed that low hsa_circ_0001675 expression did not correlate significantly with the OS of early clinical stage (I/II) patients but was related to reduced OS in advanced clinical stage (III/IV) patients (log-rank *P* = 0.0041) (**Figure 3E**). For PFS, low hsa_circ_0001675 expression was significantly associated with poor PFS in advanced T stage (T3/T4) patients (log-rank *P* = 0.0121, **Figure 3D**) and clinical stage (III/IV) patients (log-rank *P* = 0.0085 **Figure 3F**). Subsequent multivariate analysis also showed that the expression of hsa_circ_0001675 (HR:3.001, 95% CI: 1.179–7.641, *P* = 0.021) and Lymphatic metastasis (HR:0.194, 95% CI: 0.067–0.560, *P* = 0.002) were independent factors for poor HNC prognosis (**Table 2**). Overall, these results indicated that low hsa_circ_0001675 expression may correlate with the development of HNC and impact patient survival.

**Table 2:** Multivariate Cox regression analysis of prognostic factors affecting disease-free survival in HNC patients.

Variables	Multivariate analysis		
	HR	95% CI	*P*
Age (≥ 60y *vs*. <60y)	0.954	0.901–1.010	0.108
Smoking behavior (Yes *vs*. No)	0.947	0.293–3.068	0.928
Alcohol behavior (Yes *vs*. No)	0.908	0.277–2.976	0.873
Tumor site (Oral cavity/Oropharynx *vs*. Larynx/Hypopharynx);	0.988	0.389–2.513	0.980
Histologic grade (Moderately/Poorly *vs*. Well)	1.656	0.649–4.225	0.291
Tumor Invasion (T3/T4 *vs*. Tis/T1/T2)	1.311	0.255–6.734	0.746
Lymphatic metastasis (positive *vs*. negative)	0.194	0.067–0.560	0.002
Pathologic Stage (III/IV *vs*. I/II)	0.219	0.027–1.738	0.151
hsa_circ_0001675 expression (High *vs*. Low)	3.001	1.179–7.641	0.021

HR, hazard ratio; CI, confidence interval. P value indicates statistical significance.

### Bioinformatics Projections of hsa_circ_0001675

**Figure 4:**
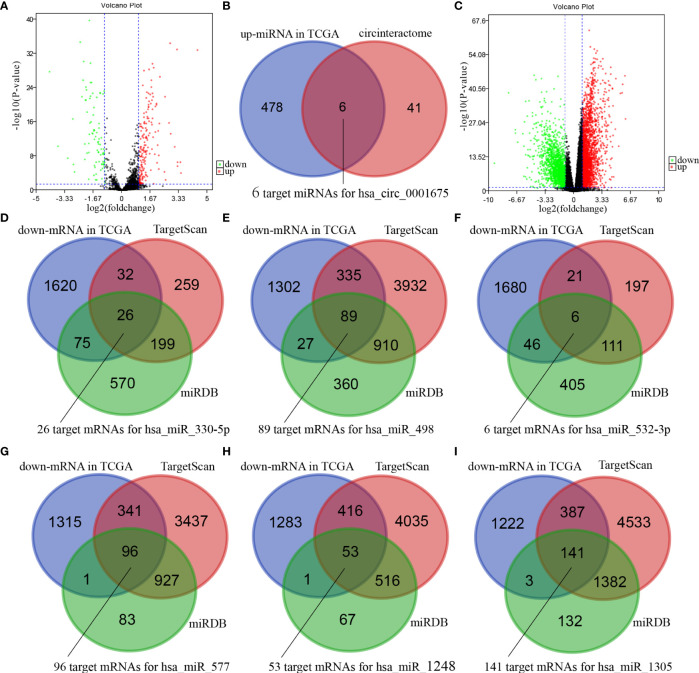
Bioinformatics projections of hsa_circ_0001675. **(A)** A volcano plot showing differentially expressed miRNAs in TCGA. The red dots indicate up-regulated miRNAs, and the green dots indicate downregulated miRNAs. The black dots represent miRNAs that were not differentially expressed. **(B)** Venn diagram of the overlap of up-regulated miRNAs in TCGA and predicted miRNAs in the circular RNA interactome database. **(C)** A volcano plot showing differentially expressed mRNAs in TCGA. The red dots indicate up-regulated mRNAs, and the green dots indicate down-regulated mRNAs. The black dots were not differentially expressed mRNAs. **(D–I)** Venn diagrams of the overlap of predicted mRNAs by TargetScan and miRDB databases and down-regulated mRNAs in TCGA.

**Figure 5:**
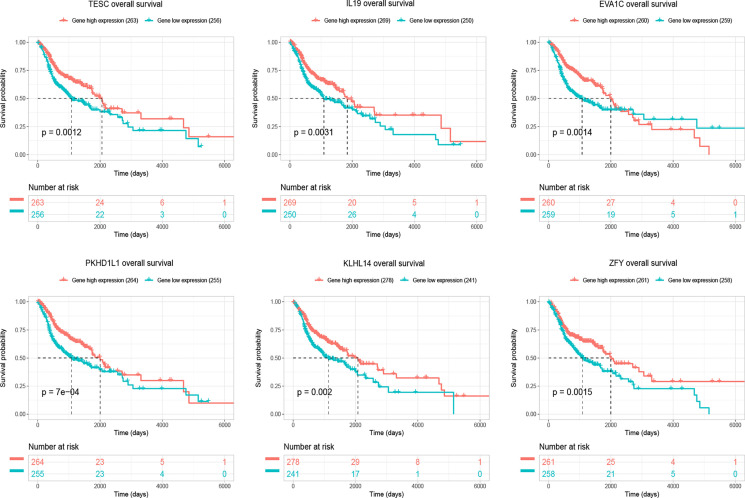
The figure showed top six target mRNAs associated with OS of HNSC patients with the most significant *P*-values in a validation set of TCGA (n=519).

Four hundred and eighty-four miRNAs that were upregulated in HNSC (*P* < 0.05, logFC >0.5) (**Figure 4A**) were screened in the TCGA database, and forty-seven target miRNAs for hsa_circ_0001675 were predicted in the circular RNA Interactome database. The intersection of the two prediction results defined six hsa_circ_0001675 target miRNAs (**Figure 4B**): hsa-miR-330-5p, hsa-miR-498, hsa-miR-532-3p, hsa-miR-577, hsa-miR-1248, and hsa-miR-1305. One thousand seven hundred and fifty-three mRNAs in the TCGA database that was downregulated in HNSC (*P* < 0.05, logFC < -1) (**Figure 4C**) were screened, and the target mRNAs of the six identified miRNAs were predicted in the TargetScan database and miRDB database. The intersection of the three prediction results was used to define the target mRNAs of miRNAs (**Figures 4D–I**). From this, 411 target mRNAs for the six identified miRNAs, hsa-miR-330-5p: 26, hsa-miR-498: 89, hsa-miR-532-3p: 6, hsa-miR-577: 96, hsa-miR-1248: 53, and hsa-miR-1305: 141, were obtained and a ceRNA network of hsa_circ_0001675 with six up expressed miRNA and 411 down expressed mRNA was constructed. Besides, 25 of the 411 mRNAs were identified as having a positive effect on the OS rate of HNSC patients in the TCGA database (*P* < 0.05) (**Table 3**), and the top six mRNAs (*P* < 0.01) were chosen for further analysis (**Figure 5**).

**Table 3:** The twenty-five predicted mRNAs that have a protective effect on the OS of HNSC (*P* < 0.05) and their circRNA-miRNA network.

circRNA	miRNA	mRNA	*P* value
hsa_circ_0001675	hsa-miR-330-5p	KLHL14	0.002
		RAB11FIP1	0.014
		SLC25A34	0.021
		FAIM2	0.039
	hsa-miR-498	ZFY	0.0015
		IL19	0.0031
		NOSTRIN	0.009
		TMPRSS11A	0.023
		GDF10	0.04
		IKZF2	0.046
		XKR4	0.047
	hsa-miR-577	TESC	0.0012
		EXPH5	0.023
		RNF222	0.037
	hsa-miR-1248	EVA1C	0.0014
		PDE4C	0.0082
		NWD1	0.0091
		FOXP2	0.035
		KLF8	0.042
		EHF	0.045
		RBM47	0.046
	hsa-miR-1305	PKHD1L1	0.0007
		MACROD2	0.0099
		ZNF662	0.038
		ZFP28	0.049

P value indicates statistical significance.

**Figure 6:**
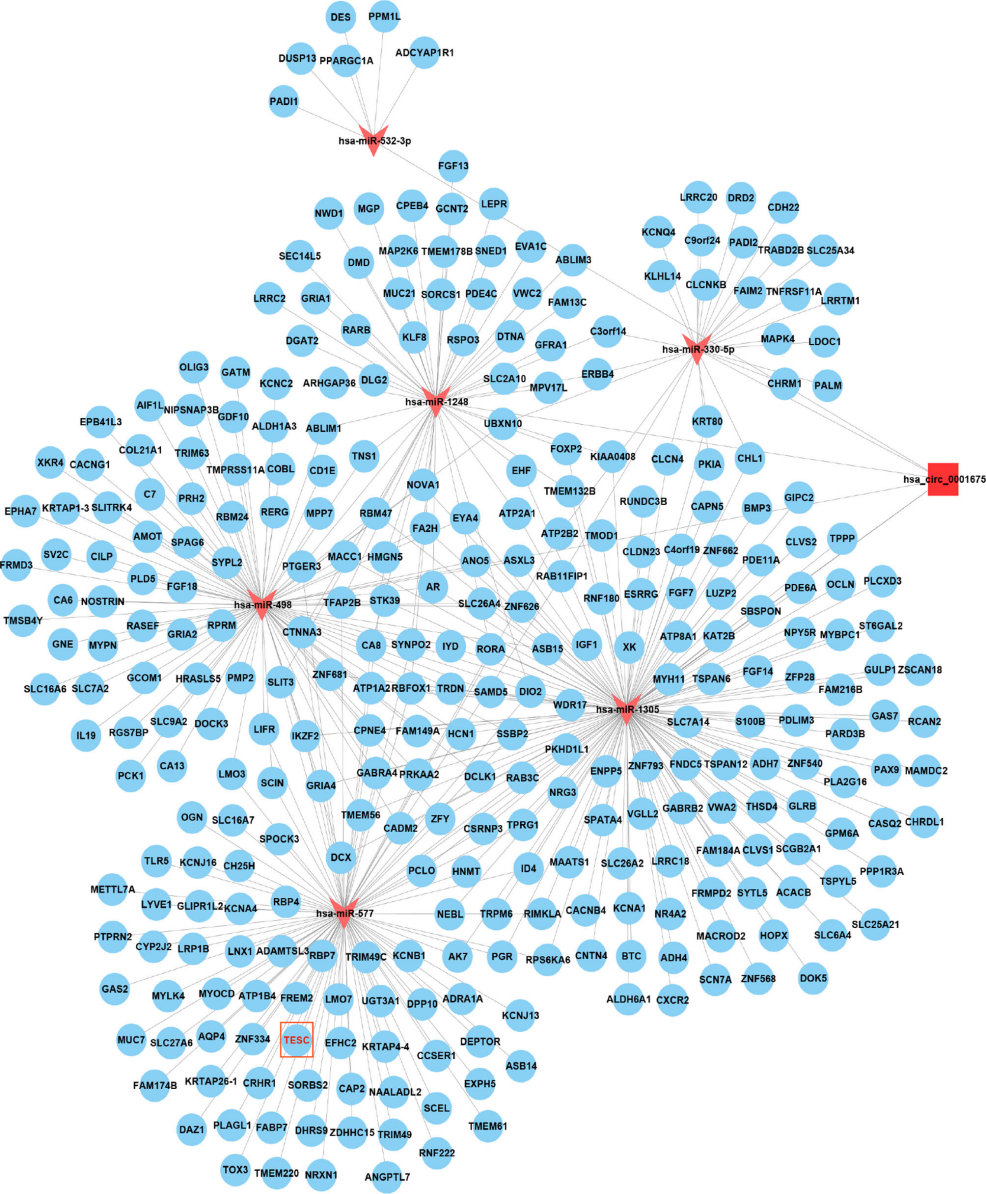
Cytoscape showed that hsa_circ_0001675 was closely related to six miRNAs, hsa-miR-330-5p, hsa-miR-498, hsa-miR-532-3p, hsa-miR-577, hsa-miR-1248, and hsa-miR-1305, and 411 mRNAs.

**Figure 7:**
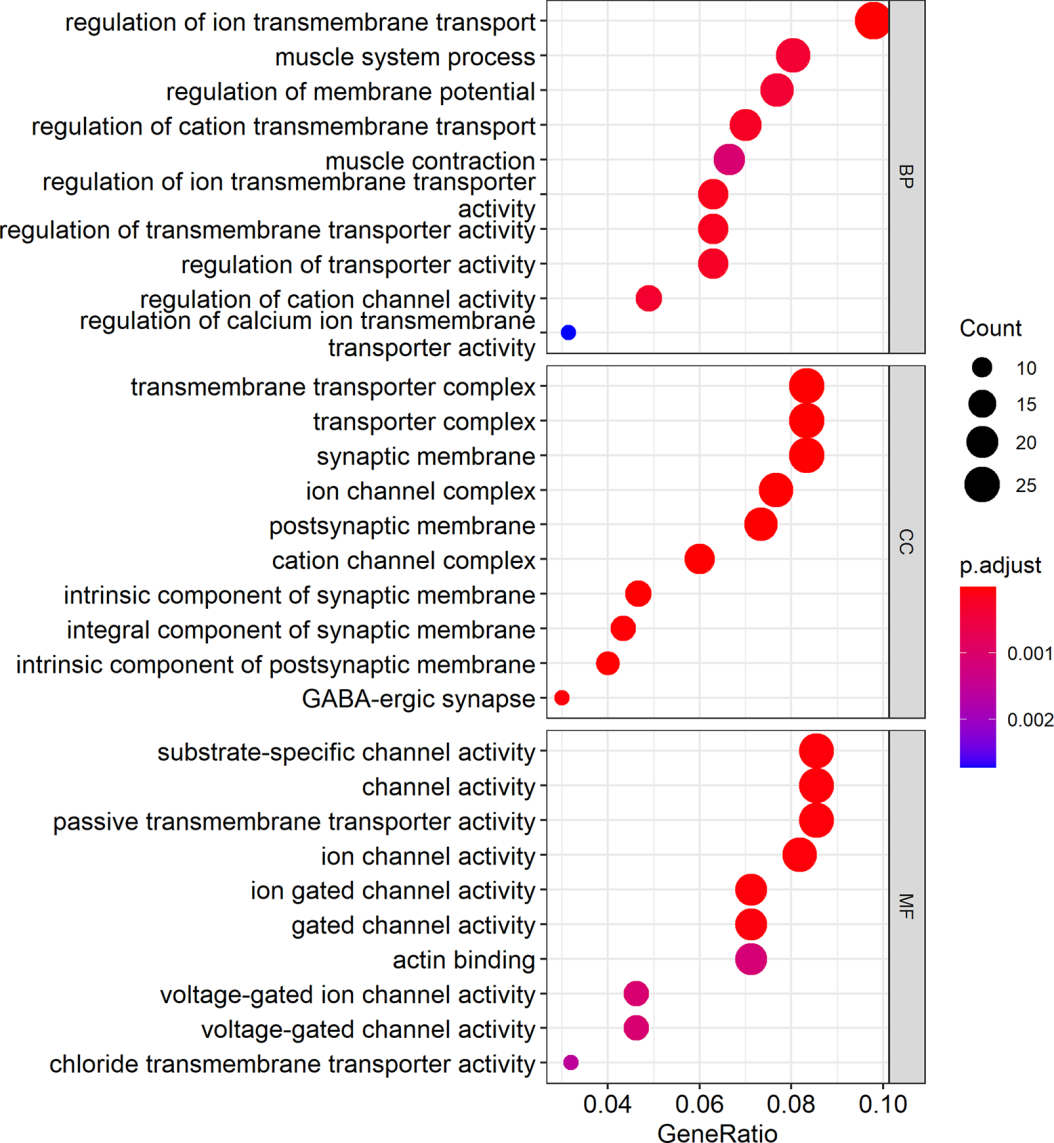
GO analysis showed the top ten biological processes (BP), molecular functions (MF), and cell components (CC). In the figure, the size of the dots indicates gene count, and the color of the dots indicates the p-value. The abscissa Generatio indicates the ratio of the number of genes mapped to GO-category to the total number of genes in the category.

**Figure 8:**
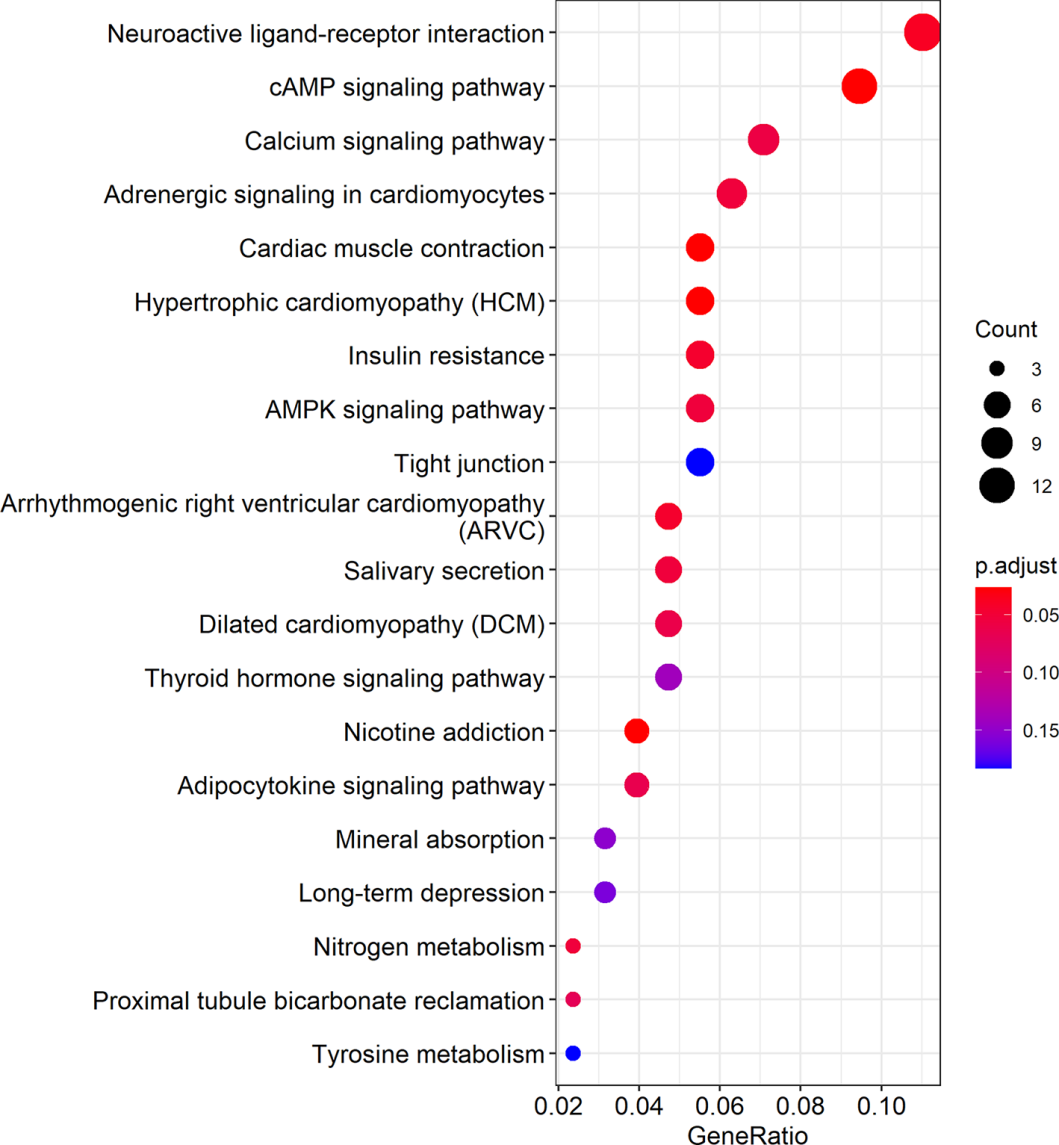
KEGG pathway analysis showed the top 20 biological pathways. In the figure, the size of the dots indicates gene count, and the color of the dots indicates the p-value. The abscissa Generatio indicates the ratio of the number of genes mapped to a KEGG pathway to the total number of genes in the pathway.

Cytoscape was used to visualize the ceRNA network (**Figure 6**). The R clusterprofiler package was used for Gene Ontology (GO) and Kyoto Encyclopedia of Genes and Genomes (KEGG) pathway analysis. GO analysis revealed that biological processes (BP), including regulation of ion transmembrane transport, muscle system processes, and regulation of membrane potential, molecular function (MF), including substrate-specific channel activity, channel activity, and passive transmembrane transporter activity, and cellular components (CC), including the transmembrane transporter complex, transporter complex, synaptic membrane, and ion channel complex were enriched (**Figure 7**). KEGG analysis indicated that the neuroactive ligand-receptor interaction was the most enriched pathway, followed by cAMP and calcium signaling (**Figure 8**).

### TESC Expression Was Reduced in HNC Tumor Tissues

**Figure 9:**
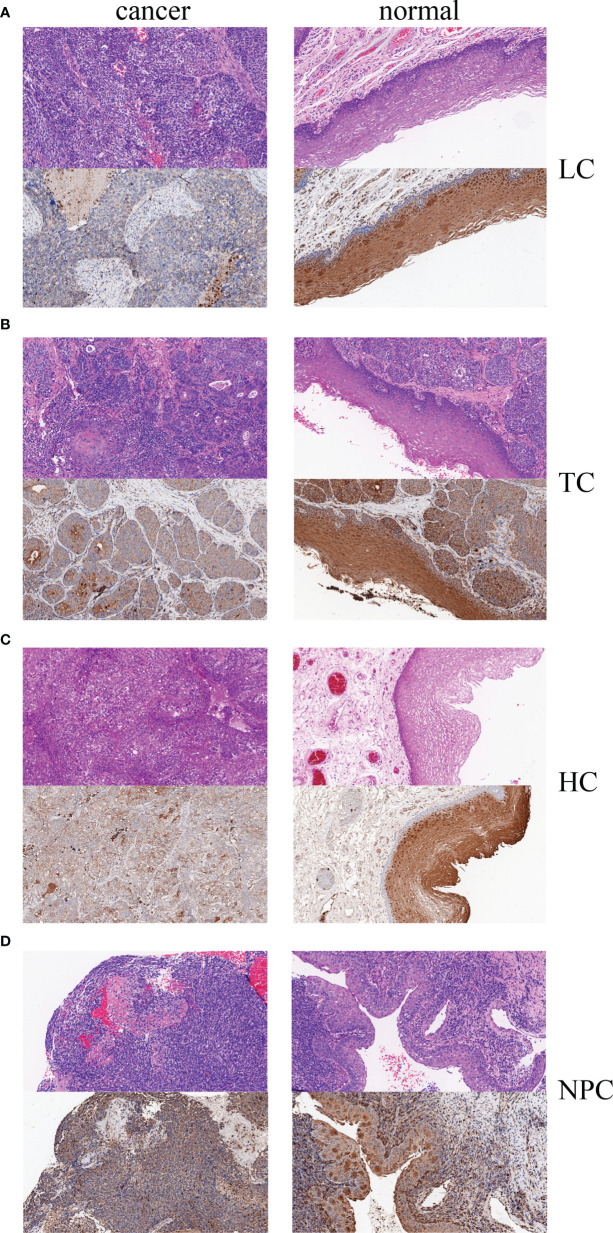
Hematoxylin-eosin (HE) staining and IHC of four HNC types, **(A)** LC, laryngeal cancer, **(B)** TC: tongue cancer, **(C)** HC, hypopharyngeal cancer, and **(D)** NPC, nasopharyngeal carcinoma.

Bioinformatics predictions indicated that tescacin (TESC) was one of the 25 identified target mRNAs, which participated in the formation of the hsa_circ_0001675/miR577/TESC signaling axis and was one of the mRNAs most closely associated with OS in HNSC patients in the TCGA database (*P* =0.0012). Thus, TESC was selected for IHC of four HNC tissue samples, tongue cancer, nasopharyngeal cancer, laryngeal cancer, and hypopharyngeal cancer, to verify its expression. The staining intensity of TESC decreased during HNC tumor progression. TESC was highly expressed in normal tissues from patients with each type of HNC, but the staining intensity was significantly lower in tumor tissues (**Figures 9A–D**). These results confirmed that TESC was differentially expressed in HNC.

## Discussion

Studies have found that non-coding RNAs play a special role in the occurrence and development of head and neck tumors, such as long non-coding RNA (lncRNA) 673 (LINC00673) was significantly up-regulated in tongue squamous cell carcinoma (TSCC) samples and was associated with poor prognosis. It can inhibit the invasion and migration ability of TSCC cells, thus participate in the progression of TSCC, and may serve as a potential biomarker for prognosis prediction and early detection of TSCC (9). lncRNAs can also interact with miRNAs to play an important protective role in TSCC (10). Therefore, non-coding RNAs are not useless fragments, and circular RNAs have great research value because of their structural specificity and biological characteristics. CircRNA was first discovered in viroid and RNA viruses more than 40 years ago (11, 12), and later found in eukaryotic cells (13). As a result of its special circular structure, circRNA has unique biological characteristics, including stability, abundance and conservation, and specificity. CircRNAs are characterized by closed loop structures without 3’ and 5’ polar ends, so are not easily degraded by exonucleases and are more stable than linear RNA (14). They are expressed in large quantities in mammals, and high-throughput sequencing has shown that there are more than one million circRNAs in human tissues, and most have highly conserved sequences across different species (15). CircRNA expression is universal, specific, and stable, and exhibits tissue-specific functions under physiological and pathological conditions (16). Our study used high-throughput sequencing technology to detect circRNA expression in tumor and adjacent tissues from HNC patients. A total of 714 differentially expressed circRNAs were detected. These circRNAs, many of which have not yet been added to circBase, enrich the circRNA family and may serve as new disease biomarkers or therapeutic targets.

The differential expression of circRNAs in tumors and adjacent tissues suggest that they may play special roles in tumors. In this study, hsa_circ_0001675 was found to be significantly downregulated in HNC tumor tissues by high-throughput sequencing and was selected for further research. Subsequent research confirmed this finding.

Experiments have confirmed that some circRNAs have important clinical significance, and even have the potential to be biomarkers. Xuan et al. (17) used microarrays to study the differentially expressed circRNAs in five pairs of laryngeal squamous cell carcinoma (LSCC) tissues and adjacent normal tissues, among which hsa_circRNA_100855 and hsa_circRNA_104912 had the most significant differential expression. Meanwhile, LSCC with higher clinical stage, T stage, or cervical lymph node metastasis had higher levels of hsa_circRNA_100855 and lower levels of hsa_circRNA_104912 expression. Furthermore, low hsa_circRNA_104912 expression correlated with poor differentiation. Based on the analysis of clinical data, we found that low hsa_circ_0001675 expression was significantly associated with tumor invasion and higher clinical stage, and Kaplan-Meier analysis showed that low expression of hsa_circ_0001675 was negatively correlated with OS and PFS rates in HNC patients, especially in advanced clinical stage (III, IV) and T stage (T3/T4), suggesting that hsa_circ_0001675 may inhibit tumor invasion and cancer development. Many tumor suppressor genes that are downregulated in head and neck cancer have been found. For example, the Microtubule-associated tumor suppressor gene (MTUS1) was found to be downregulated in OTSCC and various other cancer types and was associated with decreased overall survival (18). Considering the differential expression of hsa_circ_0001675, it may be a protective factor of HNC. The AUC of hsa_circ_0001675 was 0.7776, indicating that it had a certain diagnostic value. Future studies should consider combining hsa_circ_0001675 with other biological indicators to improve diagnostic accuracy. Multivariate analysis indicated that low hsa_circ_0001675 expression and lymphatic metastasis were independent prognostic factors.

miRNA sponge activity is the classic circRNA functional model. A miRNA is a small non-coding RNA with a length of about 22 nt which can specifically bind to the untranslated region of mRNA to regulate gene expression at the post-transcriptional level. Competing endogenous RNAs (ceRNAs) contain miRNA response elements (MREs), which can prevent miRNA inhibition of target gene expression by binding to and absorbing miRNAs, acting as an miRNA sponge (19). For example, the circRNA ciRS-7 (Cdr1as), which is highly conserved and abundant in mouse brain tissue, can become a molecular sponge for miR-7, and inhibit its function by competitively binding miR-7 and affecting its downstream gene expression (20, 21). Studies have also shown that CircHIPK3 can bind to nine different miRNAs, including miR-124, using 18 potential binding sites (22). Ma et al. (23) found that upregulation of circRNA_ACAP2 inhibited the proliferation and migration of HNSC cells, while downregulation of circRNA_ACAP2 had the opposite effect. Further research found that circRNA_ACAP2 specifically bound to miR-21-5p and inhibited its function, thereby regulating the direct target gene STAT3 of miR-21-5p, which proved that circRNA_ACAP2 functioned as a tumor suppressor gene in HNSC and was adjusted by miR-21-5p/STAT3 signaling axis regulation. These studies define the molecular mechanisms of circRNAs in HNC carcinogenesis and development, which aids the discovery of early diagnostic biomarkers and effective therapeutic targets.

The TCGA database is increasingly used for screening and testing differentially expressed genes (24). To find potential suppressing mechanism of hsa_circ_0001675, bioinformatics analysis was used to predict and identify six target miRNAs of hsa_circ_0001675, including hsa-miR-330-5p, hsa-miR-498, hsa-miR-532-3p, hsa-miR-577, hsa-miR-1248, and hsa-miR-1305. Each of these has potential binding sites on hsa_circ_0001675 and upregulated expression in HNSC in the TCGA database, suggesting that they are likely to be regulated by hsa_circ_0001675, which is downregulated in HNSC. circRNA can participate in the process of tumor as the mechanism of ceRNA, so it can become a potential tumor therapy target. In a study on the relationship between miRNAs and cancer-related signaling pathways in site-specific colorectal cancer (CRC), hsa-miR-330-5p was differentially expressed in CRC and engaged in TGF-β signaling (25). hsa-miR-498 was enriched in human melanoma exosomes and was shown to regulate TCR signaling and TNFα secretion and contribute to tumor immune escape, indicating that it has the potential to become a therapeutic target (26). Ha et al. (27) constructed a miRNA-mRNA interaction network by analyzing miRNA chips related to renal clear cell carcinoma (CCRCC) from the Gene Expression Omnibus (GEO) database. hsa-miR-532-3p and its target gene, ETS1, were shown to be significantly related to the OS of CCRCC patients. Yang et al. (28) reported that hsa-miR-577 could be competitively adsorbed by hsa_circRNA_0007334 to enhance collagen type I alpha 1 chain (COL1A1) expression and function in pancreatic ductal adenocarcinoma (PDAC). Using RNA sequencing analysis, RNA pull-down studies and dual-luciferase reporter assays, Su et al. (29) showed that hsa-miR-1305 was sponged by circRIP2 to upregulate TGFβ2 in bladder cancer through the TGFβ2/Smad3 pathway. The current study identified 411 target genes of miRNAs and constructed a ceRNA network of hsa_circ_0001675 with six up expressed miRNAs and 411 down expressed mRNAs. Several studies have determined the relationship between gene expression levels and prognosis by analyzing RNA expression data and clinical information downloaded from TCGA, and obtained genes significantly associated with overall survival time and used them to develop effective predictive models (30, 31). After screening in TCGA, twenty-five of 411 mRNAs were found to be related to OS in HNSC (*P* < 0.05), and the top six target mRNAs (*P* < 0.01) were selected for further research. These mRNAs represent potential targets for further research into their molecular mechanisms in HNSC. Cytoscape was used to visualize the circRNA-miRNA-mRNA ceRNA regulatory network. GO analysis showed that biological processes (BP) like ion transmembrane transport, muscle system processes, membrane potential regulation, molecular functions (MF) like substrate-specific channel activity, channel activity, and passive transmembrane transporter activity, and cell components (CC) like the transmembrane transporter complex, transporter complex, synaptic membrane, and the ion channel complex were enriched. KEGG analysis revealed that the neuroactive ligand-receptor interaction was the most enriched pathway, followed by the cAMP and calcium signaling pathways.

We finally constructed a ceRNA network: hsa_circ_0001675 may act as a miRNA sponge to competitively bind six target miRNAs and regulate the expression levels of 411 downstream target mRNAs. We predicted that when hsa_circ_0001675 was down-regulated in HNC, the function of its miRNA sponge was inhibited, resulting in increased expression of downstream miRNA, and finally inhibiting the expression level of mRNA, affecting the occurrence and development of HNC. Among them, TESC was the target mRNA with the most associated with the prognosis of HNC, which may play a protective role in HNC through the hsa_circ_0001675/miR577/TESC signaling axis. In this study, we first found that hsa_circ_0001675 may act as a protective factor in HNC through the miR577/TESC axis. However, TESC was found to be highly expressed in cholangiocarcinoma and mediate tumor progression by promoting the TGF-α/EGFR-FOXM1 axis, which is different from our predicted expression model (32). In fact, a gene can have different roles in different cancers, such as long non-coding RNA MALAT1 is up-regulated in non-small cell lung cancer, Breast Cancer, Osteosarcoma, is associated with poor prognosis, and promotes cancer progression (33–35). In contrast, MALAT1 is down-regulated in Endometrial Cancer, is associated with poor prognosis, and is a tumor protective factor (36). In addition, due to differences in regulatory mechanisms, a gene can have different functions in the same organ carcinomas. In colorectal cancer, inhibitors of DNA-binding protein 4 (ID4) can promote tumorigenesis by targeting brain-derived neurotrophic factor (BDNF) (37), and can also inhibit tumor growth and metastasis by inhibiting PI3K/AKT pathway and inhibiting epithelial-mesenchymal transition (EMT) in a CK18-Related manner (38). Therefore, the hsa_circ_0001675/miR577/TESC pathway in HNC needs to be experimentally verified, and the specific mechanism of TESC needs further study. IHC results showed that the staining intensity of TESC protein was significantly stronger in the adjacent tissues of four HNC types, tongue cancer, nasopharyngeal cancer, laryngeal cancer and hypopharyngeal cancer, than in the tumor tissues. The result suggested that the expression of TESC was down-regulated in HNC tissues, which preliminarily verified the possibility of the signal axis. However, the reason for the down-regulation of hsa_circ_0001675 is not yet clear, and some studies have shown that it may be related to the methylation of the gene (39, 40), and further research is needed. In addition, gene expression signatures such as loss of heterozygosity (LOH), copy number variations (CNVs), significantly up- and down-regulated genes and somatic variants (mutations and indels) can be integrated with clinical factors to discover some key genes (41). Of course, this study also has shortcomings: we have made bioinformatics prediction and simple verification of the regulatory mechanism of hsa_circ_0001675, while the expression levels and interactions of target miRNAs and target mRNAs need to be further confirmed by experiments. The tissue samples used in this study were obtained from HNC and normal adjacent tissues. Additional studies are needed to measure circRNA expression in saliva, blood, plasma, or serum and to assess its relationship with HNC.

## Conclusion

This study showed that hsa_circ_0001675 was significantly downregulated in HNC tissues and was an independent factor of HNC patients. Findings indicated that hsa_circ_0001675 has the potential to be an effective marker for the early diagnosis of HNC. In HNSC, hsa_circ_0001675 may inhibit tumorigenesis through a ceRNA network that includes six differentially expressed miRNAs and 411 differentially expressed mRNAs. In the prediction results, TESC was one of the mRNAs most closely associated with the OS of HNC patients. IHC further confirmed that TESC had reduced expression in four HNC types, suggesting that hsa_circ_0001675 may suppress HNC progression through the miR577/TESC signaling axis.

## Data Availability Statement

The raw data supporting the conclusions of this article will be made available by the authors, without undue reservation.

## Ethics Statement

The studies involving human participants were reviewed and approved by Human Research Ethics Committee of Ningbo University. The patients/participants provided their written informed consent to participate in this study.

## Author Contributions

Conceived and designed the experiments: DY, ZS. Performed the experiments and analyzed the data: YC. Contributed materials/analysis tools: ZL, QL, HR. All authors contributed to the article and approved the submitted version.

## Funding

This work was supported by grants from the Zhejiang Provincial Natural Science Foundation of China (LY19H160014, LQ21H130001), Ningbo Health Branding Subject Fund (No. PPXK2018-02), Medical and Health Research Project of Zhejiang Province (2019ZD018, 2021KY307), Ningbo Natural Science Foundation (202003N4239), Ningbo “Technology Innovation 2025” Major Special Project (2020Z097, 2018B10015). Basic Research Program of Hunan Health Commission (20201650), Zhejiang Provincial Natural Science Foundation of China (LY20H130001).

## Conflict of Interest

The authors declare that the research was conducted in the absence of any commercial or financial relationships that could be construed as a potential conflict of interest.

## Publisher’s Note

All claims expressed in this article are solely those of the authors and do not necessarily represent those of their affiliated organizations, or those of the publisher, the editors and the reviewers. Any product that may be evaluated in this article, or claim that may be made by its manufacturer, is not guaranteed or endorsed by the publisher.
